# A Bioinspired Approach to Next-Generation Vaccines in Solid Tumors with Engineered Cell Membranes

**DOI:** 10.34133/research.1193

**Published:** 2026-03-20

**Authors:** Weiyue Zhang, Maike Chen, Xin Huang

**Affiliations:** ^1^ Department of Endocrinology, Union Hospital, Tongji Medical College, Huazhong University of Science and Technology, Wuhan 430022, China.; ^2^ Department of Orthopaedics, Union Hospital, Tongji Medical College, Huazhong University of Science and Technology, Wuhan 430022, China.; ^3^ Hubei Key Laboratory of Regenerative Medicine and Multi-disciplinary Translational Research (Huazhong University of Science and Technology), Wuhan, Hubei 430022, China.

## Abstract

In solid tumors, the immunosuppressive tumor microenvironment and antigenic heterogeneity pose important challenges for effective immunotherapy, often leading to limited T-cell infiltration and inadequate immune activation. To overcome these barriers in solid tumors, the development of next-generation vaccines capable of eliciting robust and durable anti-tumor immunity has constituted a major focus in the clinic. A promising strategy involves a bioinspired approach that functionalizes synthetic nanocarriers with native cell membranes. These engineered platforms are designed to preserve the surface properties of native cell membranes (immune cells, nonimmune cells, and hybrid cell membranes) to enhance antigen presentation, prolong systemic circulation, and improve biocompatibility. This study systematically examines the design principles and mechanisms of bioinspired vaccines with native cell membranes, highlighting their capability to integrate multiple antigenic and adjuvant signals for superior antigen presentation and T-cell activation. We further explore the synergistic therapeutic effects of the next-generation vaccines when combined with common anti-tumor therapies. Moreover, the primary challenges for their clinical translation in solid tumors are critically discussed. These bioinspired nanoplatforms represent a transformative direction for developing more effective and personalized immunotherapies for solid tumors.

## Introduction

As a formidable challenge in global healthcare, tumors are characterized by uncontrolled cellular proliferation and metastatic potential [1,2]. Tumors could be broadly categorized into hematological malignancies, which originate from blood-forming tissues, and solid tumors, which develop as distinct tissue masses [3,4]. Solid tumors constitute the majority of tumor cases worldwide and are further classified based on their tissue of origin. Unlike hematological malignancies that circulate freely, solid tumors establish complex microenvironments characterized by abnormal vasculature, hypoxic regions, and dense stromal barriers [5]. These physical and biological features create substantial obstacles for therapeutic interventions, particularly for immunotherapeutic approaches that rely on efficient immune cell infiltration and function [6]. The tumor microenvironment (TME) in solid tumors represents a critical determinant of therapeutic outcomes [7]. This specialized niche comprises not only malignant tumor cells but also various stromal components including cancer-associated fibroblasts, endothelial cells, and an extensive network of immune cells [8]. The dynamic interactions within this ecosystem facilitate immune evasion through multiple mechanisms: recruitment of immunosuppressive cells such as regulatory T cells and myeloid-derived suppressor cells (MDSCs), expression of immune checkpoint molecules, and secretion of inhibitory cytokines [9–12]. Furthermore, the spatial and temporal heterogeneity of solid tumors contributes to variable antigen expression patterns, enabling immune escape and fostering therapeutic resistance [13]. To resolve the heterogeneity of solid tumors, a previous study introduced Scoring Immunological Intratumor Heterogeneity (ScImTH) as a novel algorithm that quantifies immunological intratumor heterogeneity by calculating the Shannon entropy of immune cell proportions. Across multiple datasets, a lower ScImTH score predicts unfavorable survival, an immunosuppressive microenvironment, and resistance to immunotherapy, outperforming current biomarkers. The findings position ScImTH as a promising clinical biomarker and support that loss of immune diversity is a hallmark of tumor progression [14].

The evolution of anti-tumor therapy has progressed through several distinct phases. Traditional modalities including surgery, radiation, and chemotherapy form the foundation of anti-tumor treatment, focusing primarily on direct tumor-killing effects [15,16]. Despite their efficacy in a substantial proportion of patients, these interventions are frequently associated with dose-limiting toxicity in healthy tissues and exhibit limited efficacy against metastatic disease. The emergence and broad application of omics technologies have transformed the drug development paradigm, leading to the innovative approach known as network pharmacology. Moving forward, the integration of network pharmacology with omics is poised to play an essential role in advancing tumor drug development [17]. The novel targeted therapies represented a major advancement, allowing for more precise intervention against specific molecular alterations driving tumor growth [18]. Most recently, immunotherapy has emerged as a cornerstone of oncology, harnessing the body’s immune system to recognize and eliminate tumor cells. Immune checkpoint inhibitors [19–21] and adoptive cell therapies [22,23] have demonstrated remarkable success, particularly in hematological malignancies [24–26]. However, their efficacy in solid tumors remains limited by the immunosuppressive TME that poses a shared obstacle to other modalities [27]. Nanomedicine such as advanced nanomaterials and targeted drug delivery systems could optimize region-specific biodistribution and therapeutic efficacy, which could play a vital role in modulating both solid tumors and the TME. Nanomaterials act on the TME primarily through 4 mechanisms to improve anti-tumor treatment: metabolic interventions, targeting strategies, drug delivery nanosystems, and combination therapies. These mechanisms are frequently integrated to comprehensively remodel the TME and achieve optimal therapeutic outcomes [28].

Cancer vaccines constitute a promising branch of immunotherapy designed to activate the immune system against tumor-specific targets [10,29]. Unlike preventive vaccines, therapeutic tumor vaccines aim to stimulate preexisting immunity to eradicate established tumors and prevent recurrence [30]. The development of these vaccines has evolved from early whole-cell approaches to modern platforms utilizing defined antigens, including neoantigens derived from tumor-specific mutations [31]. Although grounded in a robust rationale, clinical success has been hindered by 2 major barriers: inefficient antigen-specific immunity and a counteractive immunosuppressive TME. Recent progress in nanotechnology has provided a versatile platform for developing next-generation vaccines [32–34]. Bioinspired approaches, which combine synthetic nanocarriers with natural biological components, represent a particularly innovative strategy [35]. Chen et al. [36] reviewed the existing strategies used in extracellular-vesicle-based tumor immunotherapy, aiming to propel the development of this emerging crucial field. These platforms can be engineered to mimic natural biological processes, enhancing their ability to navigate the physiological barriers that limit conventional vaccines [37]. By incorporating cell membrane coatings onto nanoparticle (NP) cores [38–40], researchers could develop vaccines that enable cell-specific targeting of immune cells, prolong systemic circulation, and provide more effective immune activation.

This review highlights the next-generation vaccines in solid tumors by engineering native cell membrane with nanocarriers. We further investigate the current state of bioinspired vaccines for solid tumors, exploring their design principles, underlying mechanisms, and combinatorial potential with other anti-tumor therapies. More importantly, we discuss the translational challenges facing these innovative platforms and their potential to overcome the barriers in immunotherapy for solid tumors.

## Next-Generation Vaccines in Solid Tumor

### Types of vaccines

The development of vaccines has evolved through several generations, each with distinct mechanisms and applications. Cell-based vaccines represent the foundational approach, utilizing whole tumor cells or dendritic cells (DCs) loaded with tumor antigens [41,42]. Autologous tumor cell vaccines leverage patient-derived tumor cells, providing a personalized antigen repertoire, while allogeneic vaccines use established tumor cell lines for broader applicability [43]. DC-based vaccines employ ex vivo antigen-loaded DCs to prime T-cell responses yet face challenges in standardization and scalability [44,45].

Nucleic acid vaccines have emerged as a versatile platform, encompassing both DNA and messenger RNA (mRNA) formats [46]. These vaccines encode tumor antigens and can be rapidly produced, particularly beneficial for personalized neoantigen vaccines. mRNA vaccines, packaged with lipid NPs, demonstrate enhanced safety profiles and efficient protein expression [47–50]. Viral vector vaccines utilize engineered viruses to deliver tumor antigen genes, leveraging the natural infectivity of viruses to stimulate robust immune responses. However, preexisting immunity to viral vectors may limit their repeated administration [51,52].

Peptide-based vaccines constitute another major category, employing synthetic tumor-associated peptides to elicit specific T-cell responses [53]. These well-defined antigens offer manufacturing advantages but may be constrained by human leukocyte antigen restriction and limited antigen breadth. Each vaccine platform presents unique advantages in inducing anti-tumor immunity yet faces particular challenges in addressing the complex immunosuppressive TME of solid tumors [54].

Immunoproteomics, the large-scale study of proteins involved in immune responses, is pivotal for identifying novel vaccine candidates. A previous review focuses on its methodologies and their application in vaccines, highlighting its growing promise for developing peptide-based and viruslike particle vaccines [55]. Immunoproteomics could act as a key direction for the future of vaccine design.

### The difference of vaccines in solid tumors and hematological malignancies

**Fig. 1.**
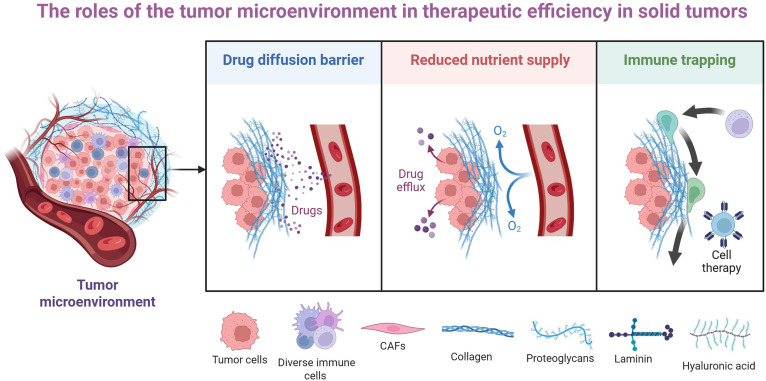
The roles of the tumor microenvironment (TME) in the therapeutic efficiency in solid tumors. The TME critically limits therapeutic efficiency in solid tumors through 3 major mechanisms. First, abnormal vasculature creates a drug diffusion barrier, preventing therapeutic agents from reaching tumor cells. Second, hypoxia and acidosis within the TME cause metabolic stress and reduced nutrient supply, which can render tumor cells less responsive to treatment. Third, the TME induces potent immune suppression and trapping by recruiting inhibitory cells and up-regulating checkpoint molecules. CAFs, cancer-associated fibroblasts.

The application of vaccine strategies differs substantially between solid tumors and hematological malignancies, primarily due to distinct microenvironments and anatomical considerations [56,57]. Solid tumors present unique challenges characterized by dense stromal barriers, hypoxic regions, and heterogeneous antigen expression in the TME (Fig. 1). The physical barrier of the extracellular matrix impedes vaccine-induced immune cell infiltration, while the immunosuppressive TME inactivates infiltrating T cells through multiple mechanisms including regulatory T cells, MDSCs, and immunosuppressive cytokines [58]. In contrast, hematological malignancies often reside in more accessible anatomical locations such as blood, bone marrow, and lymphoid organs. The absence of robust physical barriers facilitates better distribution of vaccine components and immune cell interactions. Hematological malignancies offer well-defined vaccine targets, including lymphoma-specific immunoglobulin idiotypes and leukemia-associated cancer-testis antigens [59,60]. The immune TME also differs. Hematological malignancies often arise in immune-rich environments, potentially enabling more effective engagement of immune effector mechanisms. However, they may also exploit native immunoregulatory pathways more potently. Solid tumors, particularly in advanced stages, establish profoundly immunosuppressive niches that actively exclude or inactivate T cells, necessitating complementary strategies to reverse immunosuppression [61].

These fundamental differences dictate distinct vaccine design strategies. Solid tumor vaccines require strategies to overcome physical and immunological barriers, often incorporating components that modulate the TME. Hematological malignancy vaccines can focus more directly on inducing potent antigen-specific responses against well-defined targets. Thus, although the design logic differs based on disease site characteristics, their core objective remains aligned: to achieve efficient recognition and eradication of malignancies through precise activation and guidance of the adaptive immune response.

### Bioinspired vaccines: Engineering native cell membranes with nanocarriers

**Fig. 2.**
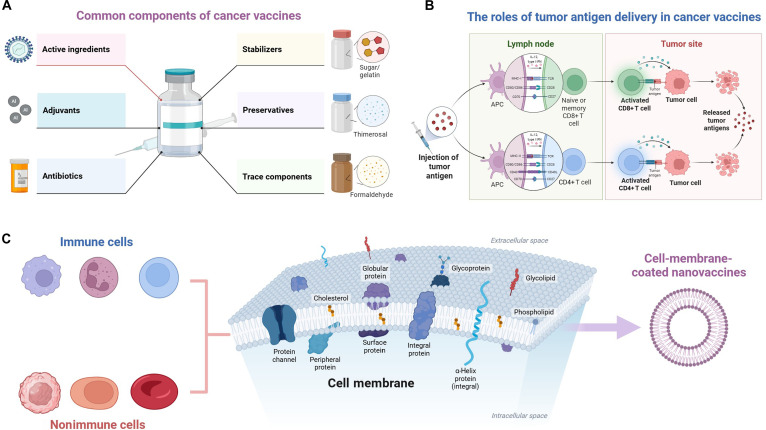
Bioinspired vaccines: engineering native cell membranes with nanocarriers. (A) Common components of cancer vaccines include active ingredients, adjuvants, and stabilizers. (B) The roles of tumor antigen delivery in cancer vaccines. This process begins with the facilitated release of tumor antigens, which are efficiently captured by antigen-presenting cells (APCs). The APCs then migrate to draining lymph nodes, where they present the processed antigens to T cells, activating and priming antigen-specific cytotoxic T cells. Ultimately, these activated T cells traffic to the tumor site, recognizing and directly killing tumor cells that express the target antigen, thereby translating vaccine administration into a potent therapeutic effect. (C) Different cell membranes derived from immune cells or nonimmune cells for the preparation of next-generation vaccines in solid tumors. IL-12, interleukin-12; IFN, interferon; MHC-I and MHC-II, major histocompatibility complex classes I and II; TCR, T-cell receptor.

Bioinspired vaccines represent a novel shift in cancer vaccine design, integrating synthetic nanocarriers with natural biological components [62]. This innovative approach involves coating NP cores with cell membranes derived from various sources, preparing hybrid platforms that preserve both the functionality of natural cell membranes and the versatility of engineered nanocarriers [63]. The strategic selection of membrane sources enables tailored functionality (Fig. 2). Tumor cell membranes present autologous tumor-associated antigens (TAAs) in their native conformation, preserving critical epitopes for immune recognition [64,65]. These membranes naturally contain endogenous signals and chaperone proteins that facilitate antigen cross-presentation. Immune cell membranes, particularly those from DCs or macrophages, offer homing molecules and costimulatory signals that enhance lymph node targeting and T-cell priming [66–68]. Erythrocyte membranes provide exceptional longevity in circulation, evading immune clearance through CD47-mediated self-recognition [69,70].

The engineering process involves several critical steps: cell membrane isolation from source cells, NP core fabrication, and cell membrane coating through extrusion or sonication methods [71,72]. The nanovaccines typically range from 100 to 200 nm, optimizing lymph node drainage and cellular uptake. NP cores can be loaded with diverse cargoes including antigens, adjuvants, cytokines, or chemotherapeutic agents, creating multifunctional systems. These bioinspired platforms demonstrate advantages in preclinical models. They enhance antigen presentation by preserving natural antigen conformation and incorporating endogenous adjuvants. The cell membrane coating provides immunological camouflage, reducing clearance and extending circulation half-life. Furthermore, they can be readily engineered as a modular platform to incorporate diverse immunostimulatory signals, thereby orchestrating a coordinated innate and adaptive immune response with spatiotemporal control [73–75]. The advantages and disadvantages of different cell membrane sources are summarized in Table 1, thereby selecting the appropriate cell membranes when different requirements arise in solid tumors. Selecting a cell membrane source involves balancing specific functional advantages, manufacturing feasibility, and clinical risk. Currently, erythrocyte membranes are the most mature for long-circulating delivery due to their superior stealth and well-established preparation. Platelet and tumor cell membranes offer unique value for specific targeting applications. Current research focuses on optimizing cell membrane fusion techniques, developing hybrid membranes combining features from different cell types [76,77], and incorporating targeting ligands for enhanced cell-specific delivery [78]. However, the most promising hybrid membrane technology is severely hindered by core challenges: unstable fusion processes, easy damage to protein function, and high barriers to scalable production [79]. Future breakthroughs depend on developing more controllable membrane fusion and characterization techniques, alongside establishing robust quality control standards to drive their clinical translation. The integration of bioinspired vaccines with other modalities, such as immune checkpoint inhibitors or conventional therapies, shows promise in overcoming immunosuppression and achieving comprehensive anti-tumor immunity. As manufacturing processes mature and our understanding of membrane biology deepens, these platforms are poised to make contributions to anti-tumor immunotherapy.

**Table 1.** The advantages and disadvantages of different cell membrane sources

Cell membrane sources	Advantages	Disadvantages
Immune cell membranes	• Strong inflammation and tumor-targeting ability • Unique biointerface functions (e.g., the neutrophil membrane can traverse the blood–brain barrier) • Immunomodulatory potential	• The membrane protein profile is highly dependent on cell state, making preparation conditions critical • Complex immunogenicity risk • Source and expansion limitations: primary immune cells are hard to expand
Tumor cell membranes	• Homologous targeting to tumor cells • Personalized vaccine potential • Immunostimulatory properties	• High immunogenicity and safety risks • Complex preparation and poor reproducibility • Challenges in standardizing personalized sources
Erythrocyte membranes	• Prolonged blood circulation • High biocompatibility and safety • Relatively simple preparation: easy to source, isolate, and purify; the membrane structure is stable	• Lacks active targeting ability • Limited functionality
Stem cell membranes	• Inflammation or tumor-targeting ability • Low immunogenicity • Pro-repair potential	• Tumorigenicity concerns • Complex culture and phenotype control • Unclear functional mechanisms: specific membrane molecules responsible for targeting and repair are not fully elucidated
Bacterial outer membranes	• Potent immune activation • Easily modified via genetic engineering to express heterologous antigens • Low cost and easy culture	• High immunogenicity and toxicity risk • Poor biocompatibility • Public acceptance and regulatory hurdles
Hybrid cell membranes	• Function integration and synergy • Designable performance: carrier properties can be customized by adjusting membrane ratios • Overcomes single-source limitations	• Complex and unstable preparation process • Low membrane protein function retention • Extremely difficult to scale up: complex process, poor reproducibility, lack of standardized quality control methods

In summary, bioinspired vaccines represent a paradigm shift in cancer vaccine design by integrating synthetic nanocarriers with natural biological membranes. However, their clinical translation, particularly for the most promising hybrid membrane technology, remains constrained by core challenges such as unstable fusion processes and scalability. Future advancement hinges on interdisciplinary collaboration among materials science, immunology, and process engineering to overcome these technical barriers and enable synergistic integration with other therapeutic modalities.

## Bioinspired Vaccines with Immune Cell Membranes in Solid Tumor

**Fig. 3.**
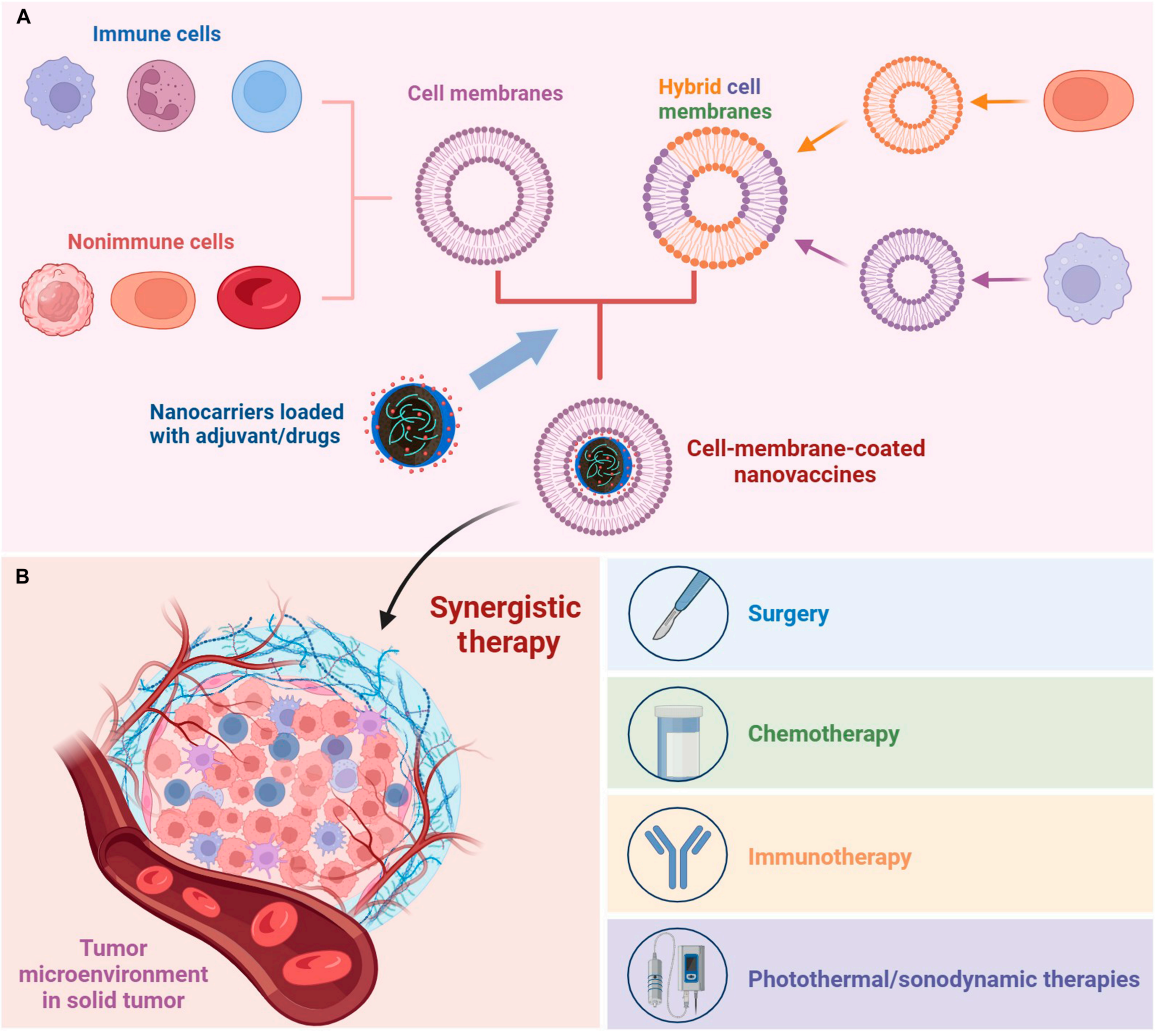
Schematic overview of next-generation vaccine preparation and synergistic therapy integration in solid tumors. (A) The preparation involves creating a biomimetic core–shell nanostructure. First, nanocarrier cores loaded with adjuvants/drugs are synthesized. Separately, cell membranes (immune cells, nonimmune cells, or hybrid cell membranes) are harvested and purified. These membranes are then fused onto the core surfaces via coextrusion, sonication, or microfluidic electroporation, forming a stabilized, antigen-presenting vaccine. (B) For synergistic therapy, these engineered nanovaccines are designed to combine with conventional treatments. For instance, they can be co-administered with chemotherapy to induce immunogenic cell death (ICD) and release more antigens, combined with immune checkpoint inhibitors to reverse immunosuppression, or integrated with photothermal therapy where the vaccine’s core provides localized heat to ablate tumors, while the membrane shell triggers a systemic immune response against residual tumor cells.

Bioinspired vaccines represent an advanced strategy for solid tumor immunotherapy [80–82]. Their core–shell architecture integrates an NP core, coloaded with tumor antigens and adjuvants, enveloped by functional cell membranes (Fig. 3A). The use of immune cell membranes confers inherent advantages for vaccine design, leveraging their native biological composition and functions [83]. This critical immune cell membrane coating serves multiple essential roles: it provides innate targeting tropism to lymphoid organs and antigen-presenting cells (APCs) such as DCs and macrophages, enhances systemic biocompatibility, and actively engages in immune signaling (Table 2). By leveraging these native membrane properties, the vaccine efficiently initiates a coordinated immune activation process. This includes enhanced antigen uptake and processing by APCs, the subsequent priming of effector T cells, and, ultimately, the establishment of potent and durable anti-tumor immunity.

**Table 2.** The immune cell-membrane-coated vaccines in solid tumors

Cell membrane types	Nanocarriers	Antigen	Adjuvant/drugs	Tumor cell lines	Solid tumors	References
Proinflammatory leukocytes membranes	EYLN	LFA-1	Dox, siLPCAT1	KYSE-150 cells	Esophageal tumor	[84]
DC membranes	Mesoporous silica framework	H22-specific neoantigen	Captoprils, Fe(III)	H22 cells	Hepatocellular carcinoma	[92]
Bone-marrow-derived DC membranes mixed with TEXs	Self-assembled ferrous ion NPs	TAA	NA	GL261 cells	Glioma	[94]
M1 macrophage membranes	shRNA-PEI-iRGD	NA	shRNA-Ptpn2 system combined with Dox	B16F10 cells	Melanoma	[95]

EYLN, egg yolk lipid nanovector; LFA-1, lymphocyte function-associated antigen 1; Dox, doxorubicin; siLPCAT1, LPCAT1 small interfering RNA; siRNA, small interfering RNA; NPs, nanoparticles; TAA, tumor-associated antigen; DC, dendritic cell; NA, not applicable; TEXs, tumor-derived exosomes; shRNA, short hairpin RNA; Ptpn2, protein tyrosine phosphatase nonreceptor type 2; PEI, polyethylenimine; iRGD, internalizing RGD

### Proinflammatory leukocyte membranes

Proinflammatory leukocyte membranes offer distinct advantages in cancer vaccine design due to their unique biological functionalities. Unlike synthetic coatings, these native membranes possess inherent targeting capabilities through surface adhesion molecules like lymphocyte function-associated antigen 1 (LFA-1), which enables precise delivery to lymphoid tissues and APCs. This targeting specificity enhances cellular uptake and antigen processing efficiency. Moreover, these membranes provide active immunomodulation through their preserved costimulatory molecules and cytokine receptors. They directly facilitate DC maturation and T-cell activation, creating a more robust anti-tumor immune response. The membranes also demonstrate improved biocompatibility and extended circulation time, while simultaneously helping to counteract the immunosuppressive TME. For instance, one study developed a novel bioinspired vector (mEYLNs-Dox/siLPCAT1) by coating a lipid-based NP with leukocyte membranes for targeted codelivery of doxorubicin (Dox) and LPCAT1 small interfering RNA to esophageal tumors [84]. The leukocyte membrane coating enabled enhanced cellular uptake through LFA-1-mediated targeting, inhibiting tumor proliferation and metastasis while demonstrating improved pharmacokinetic profiles and tumor suppression in vivo, representing a promising combined chemo-gene therapy strategy for esophageal cancer.

This multifunctional platform represents an important advancement over conventional vaccine strategies, addressing critical challenges in targeted delivery, immune activation, and TME modulation. The unique combination of biological targeting, immune signaling, and biocompatibility makes proinflammatory leukocyte membranes particularly valuable for developing next-generation cancer immunotherapies against challenging solid tumors.

### DC membranes

Neoantigens, which arise from tumor-specific somatic mutations, have gained attention as ideal targets for cancer vaccine development. These antigens offer exceptional advantages including minimal off-target effects, high immunogenicity, and the absence of preexisting immune tolerance [85,86]. Supported by this rationale, neoantigen-loaded DC vaccines have demonstrated the ability to activate robust T-cell responses against tumors with favorable safety profiles, as evidenced in numerous preclinical and clinical trials [87]. Nevertheless, the clinical efficacy of these vaccines remains limited when used alone, highlighting the need for combination strategies to enhance their therapeutic potential [88]. Moreover, live cell-based vaccines face limitations including a short shelf life, vulnerability in immunosuppressive conditions, and inefficient lymph node delivery. To address these challenges, the use of modified cell membranes has emerged as a promising alternative strategy [89]. DC membranes have emerged as a prominent biomaterial for tumor vaccine design. Leveraging the natural lymph node homing propensity of DCs, DC-membrane-coated vaccines demonstrate enhanced lymph-node-targeting capability, a key advantage for efficient immune activation [90]. Furthermore, these membrane-coated vaccines facilitate improved antigen uptake, processing, and presentation by immune cells, thereby amplifying the subsequent anti-tumor immune response [91].

One previous study developed an innovative DC-membrane-coated nanovaccine for hepatocellular carcinoma treatment [92]. The vaccine features a mesoporous silica core coloaded with the photosensitizer SiPcCl_2_ and Fe(III)–captopril complexes. The Fe(III)–captopril component reprograms protumoral N2 neutrophils to anti-tumoral N1 phenotype, suppressing tumor growth through angiogenesis inhibition. Simultaneously, photodynamic therapy could induce immunogenic cell death (ICD) and rapid antigen release, effectively creating an in situ tumor vaccine. This approach demonstrates dual functionality: enhancing lymph node homing to activate CD8+ T cells directly while converting immunologically “cold” tumors to “hot” tumors through robust T-cell infiltration [93]. The study reveals a concerted multimechanism approach that synergizes neutrophil polarization with enhanced antigen presentation and T-cell activation for potent anti-tumor immunity. In another study, a vaccine was developed by integrating radiotherapy-induced immunogenic antigens and tumor-derived exosomes with bone-marrow-derived DC membranes [94]. This membrane-based complex retained functional molecules from mature DC, including costimulatory proteins CD80/CD86, major histocompatibility complex class I (MHC-I) antigen complexes, and the CC-chemokine receptor 7 (CCR7), enabling precise antigen delivery to lymph nodes and potently enhancing the presentation process.

### Macrophage cell membranes

Macrophage membrane coating confers advantages for nanodrug delivery, including prolonged circulation time, efficient tumor targeting through innate chemotaxis, and unique access to avascular tumor regions. In a representative study, Yang et al. [95] developed M1-macrophage-membrane-coated NPs coloaded with Dox and shRNA-Ptpn2, which enhanced tumor accumulation while evading reticuloendothelial clearance. Similarly, another study created a macrophage-membrane-camouflaged Bacillus Calmette–Guérin platform that induced trained immunity in tumor-associated macrophages and improved targeting in Lewis lung carcinoma models [96]. These approaches demonstrate how macrophage membrane coating enhances both the pharmacokinetics and therapeutic efficacy of anti-tumor agents.

In summary, bioinspired vaccines that utilize immune cell membranes such as those from proinflammatory leukocytes, DCs, or macrophages offer a novel platform to enhance lymph node targeting, antigen presentation, and immune activation. By preserving the native homing molecules and signaling functions of these cells, the vaccines can more effectively initiate and sustain potent anti-tumor immune responses. This design represents a promising direction for overcoming the delivery and immunosuppressive barriers in solid tumor immunotherapy.

## Bioinspired Vaccines with Nonimmune Cell Membranes in Solid Tumor

Bioinspired vaccines utilizing nonimmune cell membranes, particularly those derived from tumor cells, leverage natural homotypic binding properties to achieve enhanced site-specific accumulation within solid tumors. These membranes present a comprehensive repertoire of endogenous tumor antigens that enable the immune system to recognize diverse tumor signatures and overcome the limitations of single-antigen vaccines. When further engineered with immunomodulatory molecules, such platforms can concurrently disrupt immunosuppressive networks and activate systemic anti-tumor immunity, providing a versatile therapeutic strategy for immunologically cold tumors. We summarize the most common nonimmune cell membranes in solid tumors as follows (Table 3).

**Table 3.** The nonimmune cell-membrane-coated vaccines in solid tumors

Cell membrane types	Nanocarriers	Antigen	Adjuvant/drugs	Tumor cell lines	Solid tumors	References
Tumor cell membranes	PLGA	TAA	Anti-CD40 single-chain variable fragment	Panc02 cells	Pancreatic tumor	[100]
Tumor cell membranes	PLGA	TAA	STING agonist	B16-OVA cells	Melanoma	[102]
Tumor cell membranes	PLGA	CVB3	miR-CVB3	4T1 cells	Triple-negative breast cancer	[103]
Tumor cell membranes	NA	NA	CpG oligonucleotide, anti-CD40 antibodies	4T1-OVA cells	Triple-negative breast cancer	[104]
Tumor cell membranes	G5 PAMAM dendrimers	NA	CpG oligonucleotide, ICG, toyocamycin	FAT 7 cells	Nasopharyngeal carcinoma	[105]
Tumor cell membranes	PLGA	NA	R848, Dox	4T1 cells, B16-F10 cells	Triple-negative breast cancer, melanoma	[106]
Erythrocyte membranes	PLGA	TAA	Mannose	B16 cells	Melanoma	[109]
Erythrocyte membranes	Liposomes	iPSC protein	Mannose	B16 cells	Melanoma	[110]
Stem cell membranes	PLGA	TAA	CpG	B16 cells	Melanoma	[111]
Bacterium-derived outer membrane vesicles	NA	NA	Surface-modifying PD-L1 antibodies (αPD-L1)	MC38-OVA cells or Pa02-OVA cells	Colon cancer or pancreatic cancer	[118]
Bacterial membrane vesicles	NA	OVA antigen	NA	B16-OVA cells	Lung metastatic malignancies	[119]
Bacterial cytoplasmic membranes	Mesoporous silica NPs	OVA antigen	Mannose	B16-OVA cells	Melanoma	[120]

PLGA, poly(lactic-*co*-glycolic acid); TAA, tumor-associated antigen; STING, stimulator of interferon genes; OVA, ovalbumin; DC, dendritic cell; PD-L1, programmed cell death protein 1; NPs, nanoparticles; CVB3, Coxsackievirus B3; PAMAM, poly(amidoamine); ICG, indocyanine green; iPSC, induced pluripotent stem cell; Dox, doxorubicin; NA, not applicable

### Tumor cell membranes

Tumor cell-membrane-coated vaccines represent a promising immunotherapy approach by leveraging a broad antigenic repertoire that includes both TAAs and tumor-specific antigens (TSAs), thereby enabling the elicitation of tumor-specific immune response [97]. This platform exploits central tolerance mechanisms to avoid immune elimination while enabling homotypic targeting for precise tumor delivery. Further enhancement of antigen presentation can be achieved through surface modification with functional molecules [98]. These advantages have facilitated the development of such vaccines across numerous solid tumors, demonstrating potential for clinical translation.

The immunogenicity of tumor antigens critically determines vaccine efficacy, particularly for antigens demonstrating strong MHC affinity. Senescent tumor cell membranes represent a promising antigen source due to their secretion of senescence-associated secretory phenotype factors, which enhance immunogenicity and promote potent anti-tumor immunity [99]. Membrane engineering further augments immune responses through strategic modifications. Li et al. [100] developed Nano-AAM/CD40, an antibody-anchored membrane vaccine incorporating anti-CD40 single-chain variable fragment into tumor cell membranes coating NP cores. This design integrates multiple immunogenic components while incorporating a CD40 agonist to overcome central immune tolerance and enhance T-cell activation. Stimulator of interferon genes (STING) pathway activation represents another potent strategy for improving DC maturation and antigen presentation [101], although clinical application remains limited by delivery challenges. Addressing this, Gou et al. [102] created bioinspired NPs coated with tumor cell membranes expressing CBP-12 peptide to target Clec9a+ DCs for STING agonist delivery. Surprisingly, STING agonists demonstrated enhanced stimulation efficacy in Clec9a+ DCs compared to tumor cells, resulting in robust DC activation, inflammatory cytokine production, and subsequent augmentation of CD8+ T-cell infiltration and cytotoxicity. Another study developed a personalized vaccine incorporating membranes from Coxsackievirus B3 (CVB3)-infected 4T1 breast cancer cells and heat-deactivated CVB3 encapsulated in poly(lactic-*co*-glycolic acid) (PLGA) NPs [103]. Multi-omics analysis revealed this design up-regulated immune activation genes while down-regulating immunosuppressive markers (programmed cell death-ligand 1 [PD-L1], B7-homolog 3 [B7-H3], and CD47) and enhancing immunostimulatory protein expression. These approaches collectively advance vaccine design through optimized antigen selection and enhanced immune activation strategies.

B lymphocytes have emerged as an important immune-regulating target. A spatiotemporally synchronized vaccine (CM-CpG-aCD40) was developed to enhance B-cell responses against triple-negative breast cancer [104]. By conjugating CpG oligonucleotides and anti-CD40 antibodies onto tumor cell membranes, the vaccine actively targets lymph nodes and engages multiple immune pathways. Through dual activation of B-cell receptors (via tumor antigens) and costimulatory signals (via CpG/toll-like receptor [TLR] and aCD40/CD40 interactions), it enhances antibody production and antigen presentation. The vaccine additionally activates DCs to prime CD8+ T cells and reprograms tumor-associated macrophages. This novel strategy could establish a foundation for advancing B-cell-focused immunotherapy. As for CpG oligonucleotides, another study developed a bioinspired photothermal vaccine for nasopharyngeal carcinoma by camouflaging multifunctional nanocomplexes with homologous apoptotic tumor cell membranes [105]. The vaccine combines photothermal therapy (PTT) and chemotherapy to effectively eliminate tumor cells while promoting DC maturation and M1 macrophage polarization through ICD and CpG adjuvant.

More importantly, Chen et al. [106] investigated the effect of preoperative chemotherapy on the efficacy of autologous tumor cell membrane antigen-based vaccines. Pre-treatment with liposomal Dox ameliorates the immunosuppressive TME and up-regulates immunological molecules on tumor cell membranes, resulting in vaccines that demonstrate superior prevention of postoperative recurrence and metastasis compared to chemotherapy alone. These findings provide valuable insights for optimizing the clinical application of tumor cell membrane vaccines.

### Erythrocyte membranes

The accelerated blood clearance is identified as a challenge in nanomedicine, where initial NP administration triggers rapid clearance of subsequent doses [107]. Erythrocyte-membrane-coated vaccines offer a distinct advantage in this context, as CD47 molecules on the membrane surface provide a “don’t eat me” signal that mitigates phagocytic clearance and extends systemic circulation [108]. Furthermore, these erythrocyte-coated NPs, while maintaining dimensions substantially smaller than those of native red blood cells, demonstrate enhanced tumor penetration capabilities by extravasating through tumor vasculature and accumulating in deeper tumor regions.

Engineering erythrocyte membranes with specific proteins or other substances is helpful to enhance their utility and improve efficiency. In the near future, individualized modification could make personal vaccines come true. A previous study designed a PLGA NP nanoplatform with erythrocyte membrane [109]. The designed delivery system utilizes a functionalized membrane to target mannose receptors on immune cells, thereby enhancing antigen recognition. By co-incorporating the adjuvant MPLA and tumor antigen hgp10025-33 into the membrane, the system potentiates antigen presentation and directs anti-tumor immunity. This synergistic approach results in suppression of tumor progression and metastasis. Zhai et al. [110] prepared a novel tumor vaccine platform utilizing induced pluripotent stem cells (iPSCs) as a broad-spectrum antigen source and engineered erythrocyte membranes as delivery vehicles. The modified lipid-expanded erythrocyte membrane vectors effectively preserved biological targeting functions while overcoming a limited drug-loading capacity. The resulting iPSC@RBC-Mlipo vaccine demonstrated splenic accumulation, elicited potent systemic anti-tumor immunity, and effectively suppressed tumor progression and metastasis in murine models, offering a promising universal approach for vaccination.

### Stem cell membranes

Stem-cell-membrane-coated vaccines leverage 2 key biological advantages: their inherent low immunogenicity enables an extended circulation time, while iPSCs naturally express broad-spectrum oncofetal antigens shared across multiple tumor types. Krishnan et al. [111] developed an iPSC-membrane-coated vaccine containing a TLR9 agonist (CpG oligonucleotide) that effectively induced DC maturation and activated cross-protective T-cell responses. In a complementary approach, Chen et al. [112] engineered mesenchymal-stem-cell-derived nanovesicles expressing aPD-L1, which demonstrated precise tumor targeting for photoimmunotherapy with minimal off-target effects. These strategies highlight the potential of stem-cell-based platforms for developing broad-spectrum vaccines through different yet complementary mechanisms of immune activation and tumor targeting.

### Bacterial outer membrane vesicles

The human microbiome has emerged as a fundamental regulator in tumor biology, profoundly influencing tumor initiation, immune modulation, and treatment outcomes [113]. The commensal microbiota plays a fundamental role in immune system development, and its strategic modulation represents a promising approach to enhancing anti-tumor immunity [114]. Bacterial outer membrane vesicles (OMVs), which are naturally released through controlled blebbing of gram-negative bacterial membranes, offer unique advantages as vaccine platforms [115]. These vesicles inherently contain multiple immunostimulatory components, including lipopolysaccharide, outer membrane proteins, and specific phospholipids, that function as pathogen-associated molecular patterns (PAMPs). This composition enables OMVs to protect encapsulated insoluble molecules from degradation while maintaining therapeutic concentrations during systemic transport. Moreover, their rich array of pattern recognition receptors allows OMVs to directly activate immune cells through the targeted delivery of immunomodulatory cargo. These properties make OMVs particularly suitable for next-generation vaccine development, combining natural immunogenicity with advanced drug delivery capabilities. OMVs demonstrate potential for immunomodulatory cargo delivery. Huang et al. [116] successfully induced anti-basic fibroblast growth factor (anti-bFGF) autoantibody production and suppressed tumor angiogenesis by displaying the full-length bFGF molecule on genetically engineered OMVs. However, their efficacy is constrained by maturation-induced uptake obstruction, wherein DC maturation triggered by OMV components attenuates subsequent lipopolysaccharide–TLR4 interactions, limiting sustained antigen presentation. To address this, Liang et al. developed OMVs loaded with αDEC205 antibodies. As DEC205 expression is up-regulated upon DC maturation, this strategy enables continued vesicle internalization by mature DCs, thereby enhancing antigen presentation and T-cell activation [117].

Lu et al. [118] developed an “Antigen Presentation Signal Enhancer” based on OMVs by surface-anchoring PD-L1 antibodies. This platform simultaneously enables antigen delivery and signal modulation: while the inherent immunogenicity of OMVs activates costimulatory signals, the blockade of the PD-1/PD-L1 pathway reshapes the immune synapse microenvironment and effectively restores CD80-mediated stimulation. As for an innovative inhalable vaccine system based on engineered bacterial membrane vesicles (BMVax), the ovalbumin (OVA) antigen was encoded into a pBAD-ClyA-OVA plasmid and transfected into *Escherichia coli*, enabling membrane surface expression of the target antigen [119]. Bacterial membrane vesicles were then isolated through an optimized procedure combining ultrasonication, freeze–thaw cycles, and high-speed centrifugation. The resulting BMVax maintained both immunostimulatory properties and antigen presentation capability, exhibiting uniform nanosized distribution and excellent stability. Following inhalation administration, BMVax activated germinal center B cells, mature DCs, and follicular helper T cells in tracheobronchial lymph nodes, establishing potent mucosal and systemic immunity against pulmonary metastases. Similarly, a study developed a DC-targeted vaccine by encapsulating an OVA antigen in manganese-doped silica NPs coated with mannose-modified bacterial membranes [120]. The vaccine enhances lymph node delivery, activates the cyclic GMP-AMP synthase (cGAS)–STING pathway through manganese ions, and potentiates anti-tumor immunity via improved antigen presentation and T-cell activation, offering a novel strategy for anti-tumor immunotherapy.

Bioinspired vaccines that employ nonimmune cell membranes broaden the strategic options for solid tumor immunotherapy. Platforms derived from sources such as tumor cells, erythrocytes, stem cells, or bacteria provide unique advantages including intrinsic tumor targeting, a broad antigen repertoire, prolonged circulation, and natural immunogenicity. By addressing the limitations of single-antigen vaccines and actively reshaping the TME, these versatile systems represent a promising approach for treating immunologically resistant tumors.

## Bioinspired Vaccines with Hybrid Cell Membranes in Solid Tumor

With the advancement of cell membrane coating technology, research has evolved from single-source membranes toward hybrid systems that combine 2 or more distinct membrane types. This strategy enables a synergistic combination of their respective advantages while compensating for individual limitations (Table 4). Wang et al. [92] created a personalized DC-mimicking vaccine (HybridDC) that enhances antigen presentation and activates anti-tumor immunity. The vaccine uses engineered DC membranes loaded with key components including CCR7, TAA peptide, tumor-derived exosome, and costimulatory molecules. Compared to existing vaccines, HybridDC shows better lymph node targeting and more effective remodeling of the TME.

**Table 4.** The hybrid cell-membrane-coated vaccines in solid tumors

Cell membrane types	Nanocarriers	Antigen	Adjuvant/drugs	Tumor cell lines	Solid tumors	References
Hybrid membranes of DC and bacterial membranes	OVA mRNA-loaded Mn_3_O_4_ NPs	OVA peptide as endogenous antigen	Mn^2+^ acts as a STING agonist	B16-OVA cells	Melanoma	[128]
Hybrid membranes of tumor cell membranes and *Escherichia coli* membranes	Polystyrene@polyethylene glycol NPs	NA	GM-CSF	LLC cells, B16-F10 cells	LLC, melanoma	[129]
DC membranes fused with bacterial *E. coli* cytoplasmic membranes and tumor cell membranes	F127 micelles	TAA, TSA	NA	B16 cells	Melanoma	[130]
Tumor cell membranes and liposome	NA	TSA	Metallo-agonist of manganese ions	B16-OVA cells	Melanoma	[156]

DC, dendritic cell; OVA, ovalbumin; mRNA, messenger RNA; NPs, nanoparticles; TAA, tumor-associated antigen; TSA, tumor-specific antigen; GM-CSF, granulocyte-macrophage colony-stimulating factor; LCC, Lewis lung carcinoma; NA, not applicable

Efficient antigen presentation by DCs serves as a fundamental requirement for successful mRNA vaccination, relying on adequate antigen translation and the presence of mature DCs [121]. Mature DCs further enhance T-cell activation through highly expressed costimulatory molecules that provide essential secondary signals [122,123]. The incorporation of adjuvants represents a well-established strategy to promote DC maturation and up-regulate these critical surface molecules [124,125]. Bacterial membranes, which are naturally enriched with PAMPs, function as potent innate immune adjuvants by enhancing antigen presentation and activating immune responses [126,127]. He et al. [128] developed an innovative mRNA nanovaccine (HM@Mn_3_O_4_-mRNA) to address poor lymphatic delivery of mRNA vaccines. The system comprises NPs encapsulating OVA mRNA within hybrid DC and bacterial membranes. This unique design enhances DC targeting through membrane homing properties while providing adjuvant activity via PAMPs. The vaccine demonstrates multiple synergistic mechanisms: mRNA translation into antigenic peptides for MHC-I presentation to CD8+ T cells, concurrent MHC-II-mediated CD4+ T-cell activation, and Mn^2+^ release that simultaneously enables STING pathway activation. Fu et al. [129] constructed a bioinspired vaccine Bio-HCP@FM-NPs, which is composed of senescent tumor cell membranes and *E. coli* cytoplasmic membrane extracts. This platform demonstrated potent anti-tumor efficacy across multiple models, synergizing effectively with anti-PD-1 therapy through granulocyte-macrophage colony-stimulating factor-mediated DC recruitment and activation. The vaccine induced robust antigen-specific immune responses dependent on T and B cells, prolonging postoperative survival and preventing tumor recurrence, highlighting its potential for personalized immunotherapy.

Similarly, Ren et al. [130] created an “ABC” hybrid membrane vaccine by combining membranes from DCs (A), *E. coli* (B), and tumor cells (C). This design addresses the limitations of single-antigen vaccines through its multicomponent structure: DC membranes enhance lymph node targeting and immune interaction, bacterial membranes provide natural adjuvant effects via PAMPs, and tumor cell membranes deliver a broad spectrum of tumor antigens such as TAAs and TSAs. Together, these elements create a comprehensive system that improves antigen presentation and immune activation against heterogeneous tumors.

Based on the intrinsic capacity of liposomes to stabilize transmembrane proteins, Chen et al. developed “tumosomes” as a bioinspired nanovesicle platform incorporating complete TSA epitopes. These nanovesicles were prepared through the hydration of liposomes with tumor cell membrane extracts followed by physical extrusion, creating a hybrid vaccine system that preserves the native conformation of tumor transmembrane proteins. The Mn-tumosomes could activate the cGAS–STING pathway through liposomal encapsulation of tumor transmembrane proteins and Mn^2+^. The Mn-tumosomes demonstrated enhanced DC uptake and lymph node targeting, eliciting potent tumor-specific CD8+ T-cell responses that suppressed tumor growth in murine models. Combined with immune checkpoint blockade, this easily fabricated and biocompatible platform shows promise for clinical translation in postoperative immunotherapy.

Despite the remarkable promise of hybrid cell membrane technology for advanced drug delivery and immunotherapy, their clinical translation is hindered by a series of interconnected technical difficulties spanning from fundamental biophysics to industrial manufacturing [131]. The most important challenge lies in achieving stable and homogeneous fusion between inherently incompatible donor membranes. The thermodynamic mismatch in lipid composition and the disruptive shear or thermal energy from standard fusion techniques (e.g., extrusion and sonication) often lead to phase separation, vesicle heterogeneity, and colloidal instability [79]. This process directly leads to the second critical problem: preserving the native functionality of cell membrane proteins. The forces that drive cell membrane fusion can denature membrane protein structures and degrade the specific ligand-binding or signaling capabilities [76,132]. Current methods often fail to distinguish between mere protein presence and preserved bioactivity. Ultimately, these molecular-scale challenges cascade into the formidable difficulties of industrial-scale preparation. Reproducibly sourcing and standardizing bulk membrane materials, scaling up the fusion process with precise control, and developing good manufacturing practice (GMP)-compatible purification and characterization protocols for these complex supramolecular structures are largely unresolved [79,131]. Therefore, advancing hybrid cell membrane technology requires a paradigm shift from demonstrating novel biological functions to systematically engineering solutions for these stability, functionality, and manufacturability constraints through interdisciplinary collaboration.

## Next-Generation Vaccines Combined with Common Anti-tumor Treatments

Chemotherapy remains a cornerstone in anti-tumor treatment, yet its efficacy is often compromised by off-target toxicity and subsequent immunosuppression. The integration of nanotechnology offers a promising strategy to overcome these limitations. Nanovaccines, with their targeted delivery capabilities, can be engineered to codeliver chemotherapeutic agents and immunomodulators precisely to tumor sites. In one representative study, a bioinspired vaccine was developed by coating PLGA NPs with fibrosarcoma cell membranes and loading them with the STING agonist 2′,3′-cyclic guanosine adenosine monophosphate (cGAMP) [133]. When combined with low-dose Dox, this platform not only induced ICD but also counteracted vaccine-induced expansion of MDSCs, thereby reprogramming the immunosuppressive TME. The sequencing of chemotherapy and vaccination has also been investigated as a means to enhance therapeutic outcomes. Preoperative administration of liposomal Dox was shown to prime the immune TME, increasing the potency of subsequently administered autologous tumor cell-membrane-based vaccines [106]. These findings collectively underscore the potential of rationally designed chemotherapy–vaccine combinations to achieve superior anti-tumor immunity through multimodal mechanisms.

Immune checkpoint blockade represents a transformative approach in cancer therapy, yet its efficacy is often constrained by the immunosuppressive TME. Innovative vaccines are now being designed to synergize with checkpoint inhibition by codelivering antigens, adjuvants, and immunomodulators. For instance, one study developed an OMV-based platform that simultaneously presents neoantigens and αPD-L1 antibodies to DCs [118]. This approach not only enhanced antigen cross-presentation but also achieved localized checkpoint blockade with a 10-fold reduction in antibody dose compared to systemic administration, thereby minimizing off-target effects. Recent advances in tumor immunotherapy have highlighted the critical role of natural killer cell immune checkpoints. A deeper understanding of the molecular networks regulating these checkpoints and their function within the TME will provide key insights to support the development of novel tumor vaccines [134]. Beyond PD-1/PD-L1 axis, the CD47– signal-regulatory protein alpha (SIRPα) “don’t eat me” pathway represents another key immune evasion mechanism. Researchers have employed CRISPR–Cas9 to generate CD47-deficient tumor cells engineered with calreticulin exposure [135]. Membranes from these cells were coated onto adjuvant-loaded NPs, creating vaccines that promote phagocytosis and cross-priming of tumor-specific CD8+ T cells. Chimeric antigen receptor (CAR) T-cell therapy has demonstrated remarkable success against hematological tumors. However, its effectiveness against solid tumors remains constrained by multiple factors, including an immunosuppressive TME and target antigen heterogeneity. To address these limitations, researchers are exploring innovative combinations of nanotechnology with CAR-T therapeutics. In one pioneering approach, scientists developed a bioinspired system by coating cisplatin-loaded PLGA NPs with membranes derived from anti-human epidermal growth factor receptor 2 (anti-HER2) CAR-T cells [136]. This design achieved extended blood residence time and improved tumor-specific accumulation while minimizing nonspecific clearance. The integration of CAR–cell membrane components with vaccine platforms might represent a promising direction for developing synergistic immunotherapy systems that combine the targeted cytotoxicity of cellular therapy with the broad immune activation capabilities of vaccination.

Photothermal (PTT) and sonodynamic therapies (SDT) represent promising physical approaches for anti-tumor treatment that can synergize effectively with vaccines. Both modalities induce ICD, releasing tumor antigens that enhance vaccine-initiated immune responses. Beyond direct cytotoxic effects, PTT promotes the release of TAAs and danger signals, thereby stimulating anti-tumor immunity. This immunogenic characteristic makes PTT an ideal partner for vaccine strategies. In one innovative approach, a bioinspired vaccine was constructed by coating zinc phosphate NPs with DC membranes and coloading a TSA with the natural photosensitizer melanin [137]. This formulation demonstrated enhanced photothermal conversion efficiency and excellent biocompatibility. Under laser irradiation, the platform effectively eliminated primary tumors while simultaneously triggering a systemic immune response. With superior tissue penetration over light-based therapies, SDT enables deep-tumor treatment while sparing normal tissues. The reactive oxygen species burst during SDT enhances antigen cross-presentation, providing a rational basis for combining SDT with vaccination. Researchers have developed a sono-activatable vaccine in which tumor membrane-functionalized NPs codeliver engineered mRNA and the sonosensitizer chlorin e6 [138]. Ultrasound application facilitates the endosomal escape of the genetic cargo, boosting antigen presentation. Another advanced system employed hybrid membranes derived from thylakoids and platelets, to coat NPs carrying a sonosensitizer and an epigenetic modulator [139]. This design enabled ultrasound-triggered immunogenic tumor killing and efficient targeting of both primary and metastatic sites, effectively creating an in situ vaccine that enhanced DC maturation and T-cell activation.

To sum up, these engineered vaccines are designed to combine with conventional treatments for synergistic therapy in solid tumors (Fig. 3B). For instance, they can be co-administered with chemotherapy to induce ICD and release more antigens, combined with immune checkpoint inhibitors to reverse immunosuppression, or integrated with PTT where the vaccine’s core provides localized heat to ablate tumors while the membrane shell triggers a systemic immune response against residual tumor cells. These synergistic strategies are designed to integrate effective local tumor control with a potent systemic anti-tumor immune response, thereby achieving cooperative eradication of solid tumors and TME alongside long-term immune surveillance.

## Discussions

Bioinspired cell-membrane-coated vaccines represent a frontier in anti-tumor immunotherapy, integrating nanotechnology with biological components to activate anti-tumor immunity. These platforms demonstrate unique advantages: they present diverse tumor antigens to prevent immune evasion, utilize patient-derived materials for personalized approaches, and allow flexible codelivery with other therapeutics. Despite these strengths, several aspects require optimization to enhance clinical efficacy. Two complementary approaches are emerging to improve antigen presentation. In situ vaccination utilizes locally administered components to trigger ICD, releasing a broad spectrum of endogenous antigens that stimulate systemic immunity [140,141]. This strategy offers advantages of universal applicability and comprehensive antigen coverage, although it faces challenges in monitoring specific immune responses. Conversely, personalized neoantigen vaccines employ bioinformatics to identify patient-specific mutations [142], creating highly targeted therapies that bypass central tolerance and minimize off-target effects [143,144]. Future progress will depend on balancing innovation with standardization, ultimately enabling the clinical realization of bioinspired vaccines’ potential in the treatment and prevention of solid tumors.

Translating these advanced platforms to clinical use faces substantial manufacturing challenges. Meeting GMP standards requires addressing critical issues in membrane sourcing and production consistency [145]. Tumor heterogeneity necessitates standardized membrane sources, potentially through established cell banks or immortalized cell lines. Achieving consistent batch-to-batch production is a major challenge for cell-membrane-coated nanovesicles [146–148]. The biological source of cell membranes (from cell lines or primary cells) naturally varies in protein and lipid content due to factors such as passage number, culture density, and nutrient levels. Implementing a novel approach is crucial to control this variability. It requires defining and monitoring critical quality attributes (CQAs) for both the cell membrane material and the final product [149,150]. Key CQAs include (a) the relative abundance of essential membrane proteins (e.g., CD47 and αvβ3 integrin), measured by quantitative flow cytometry or proteomics; (b) the size and surface charge of the isolated cell membrane nanovesicles; and (c) the coating efficiency and specific targeting ability of the final nanovesicles, verified through standardized binding assays. Setting strict acceptance criteria for these CQAs is fundamental for meeting regulatory requirements and ensuring reliable therapeutic performance. Moreover, implementing process analytical technologies for real-time monitoring and developing robust cold chain systems are essential for maintaining product stability during scale-up [151,152].

Virus inactivation process is a key regulatory requirement for biologics, necessitating proof of effective virus clearance. However, standard harsh inactivation methods can damage the delicate proteins and lipids of cell membranes [153]. Thus, a gentle, multistep strategy must be developed early. One viable approach involves (a) treating cell membrane nanovesicles with a mild detergent to inactivate enveloped viruses, followed by its careful removal, and (b) applying nanofiltration after coating to physically remove viral particles. Each step’s impact on product function must be rigorously tested. Data from these validation studies are essential for regulatory approval. Long-term immunogenicity poses a key risk upon repeated administration of these nanovesicles. The induction of antimembrane antibodies may accelerate clearance, neutralize therapeutic efficacy, or trigger adverse immune responses [154,155]. The level of risk largely depends on the cell membrane sources: homologous membranes may carry autoantigens or culture residuals, whereas heterologous membranes are generally more immunogenic. Practical mitigation strategies include preclinical assessment of antibody formation in animal models and its impact on efficacy; engineering the cell membrane to reduce immunogenicity; and monitoring antibody levels in patients during clinical trials, correlating them with pharmacokinetic changes.

Addressing these specific challenges through robust process control, dedicated viral clearance, and proactive immunogenicity management is therefore essential. This comprehensive approach is more than a regulatory hurdle; it represents the necessary pathway to transform cell-membrane-coated vaccines from a compelling laboratory discovery into a reliable clinical platform.

## Conclusions

Bioinspired vaccines represent a transformative approach in solid tumor immunotherapy by integrating natural cell membranes with synthetic nanocarriers. These sophisticated platforms leverage biologically derived coatings to enhance targeting precision, prolong systemic circulation, and improve antigen presentation. Their core–shell structure enables codelivery of TAAs, TSAs, and immunostimulatory adjuvants, effectively activating robust anti-tumor immunity while minimizing off-target effects. Despite promising preclinical results, clinical translation faces several challenges. Standardization of manufacturing processes, comprehensive safety evaluation, and optimization of functional modifications require further development. Addressing these hurdles through interdisciplinary collaboration will be crucial for advancing this innovative technology toward clinical application, potentially opening new avenues for effective solid tumor treatment and prevention.
